# Supplementary material for: Axon TRAP reveals learning-associated alterations in cortical axonal mRNAs in the lateral amygdala
Source: eLife. 2019 Dec 11;8:e51607. doi: 10.7554/eLife.51607 (PMC6924958; doi:10.7554/eLife.51607)
Supplement: Supplementary file 1. [file elife-51607-supp1.docx]

| **Supplementary File 1. RNA Quality Control Data** | | | | | | |
| --- | --- | --- | --- | --- | --- | --- |
| **Sample** | **RIN** | **Raw reads #1** | **Raw reads #2** | **% bases Q>= 30** | **Uniquely**  **mapped**  **reads %** | **Multi-**  **mapped**  **reads %** |
| TRAP control axons rep 1 | 7.8 | 29,430,720 | 29,430,720 | 94.48 | 77.47 | 19.04 |
| TRAP control axons rep 2 | 8.0 | 27,285,154 | 27,285,154 | 95.24 | 78.08 | 18.39 |
| TRAP control cortex rep 1 | 9.4 | 34,057,317 | 34,057,317 | 95.5 | 72.25 | 23.47 |
| TRAP control cortex rep 2 | 9.8 | 38,634,382 | 38,634,382 | 94.96 | 70.66 | 25.54 |
| TRAP trained axons rep 1 | 9.8 | 30,221,230 | 30,221,230 | 94.41 | 76.86 | 19.78 |
| TRAP trained axons rep 2 | 8.7 | 27,951,448 | 27,951,448 | 94.32 | 76.68 | 19.66 |
| TRAP trained cortex rep 1 | 9.9 | 37,791,175 | 37,791,175 | 94.79 | 69.93 | 25.90 |
| TRAP trained cortex rep 2 | 9.7 | 34,481,070 | 34,481,070 | 94.91 | 72.18 | 23.83 |
| Transc. control axons rep 1 | 6.4 | 35,934,968 | 35,934,968 | 93.03 | 87.30 | 10.10 |
| Transc. control axons rep 2 | 7.2 | 36,774,857 | 36,774,857 | 95.05 | 87.42 | 9.98 |
| Transc. control cortex rep 1 | 8.7 | 36,067,046 | 36,067,046 | 94.00 | 88.01 | 9.65 |
| Transc. control cortex rep 2 | 8.7 | 33,261,134 | 33,261,134 | 93.84 | 87.79 | 9.78 |
| Transc. trained axons rep 1 | 9.6 | 37,890,759 | 37,890,759 | 94.16 | 88.04 | 9.63 |
| Transc. trained axons rep 2 | 8.8 | 39,793,039 | 39,793,039 | 94.02 | 87.81 | 9.63 |
| Transc. trained cortex rep 1 | 8.6 | 31,509,058 | 31,509,058 | 93.81 | 88.15 | 9.42 |
| Transc. trained cortex rep 2 | 9.0 | 31,031,259 | 31,031,259 | 95.58 | 87.72 | 9.61 |
| YFP_IP control axons rep 1 | 7.0 | 39,073,113 | 39,073,113 | 94.15 | 74.32 | 21.75 |
| YFP_IP control axons rep 2 | 9.0 | 32,214,031 | 32,214,031 | 94.25 | 72.90 | 22.99 |
| YFP_IP control cortex rep 1 | 8.8 | 27,039,569 | 27,039,569 | 93.57 | 76.52 | 19.51 |
| YFP_IP control cortex rep 2 | 9.3 | 27,888,237 | 27,888,237 | 93.17 | 73.15 | 22.23 |
| YFP_IP trained axons rep 1 | 9.0 | 27,119,148 | 27,119,148 | 92.58 | 74.22 | 21.69 |
| YFP_IP trained axons rep 2 | 8.4 | 29,286,890 | 29,286,890 | 95.23 | 73.60 | 22.19 |
| YFP_IP trained cortex rep 1 | 9.5 | 30,180,396 | 30,180,396 | 94.74 | 76.00 | 19.55 |
| YFP_IP trained cortex rep 2 | 8.9 | 29,087,509 | 29,087,509 | 93.94 | 74.33 | 21.60 |
| YFP transc. control axons rep 1 | 9.5 | 32,819,895 | 32,819,895 | 94.16 | 88.17 | 9.33 |
| **Table S1. RNA Quality Control Data, cont.** | | | |  |  |  |
| **Sample** | **RIN** | **Raw reads #1** | **Raw reads #2** | **% bases Q>= 30** | **Uniquely**  **mapped**  **reads %** | **Multi-**  **mapped**  **reads %** |
| YFP transc. control axons rep 2 | 9.4 | 32,118,423 | 32,118,423 | 94.29 | 86.84 | 10.52 |
| YFP transc. control cortex rep 1 | 9.6 | 29,502,761 | 29,502,761 | 93.81 | 87.73 | 9.71 |
| YFP transc. control cortex rep 2 | 7.6 | 30,411,787 | 30,411,787 | 93.38 | 87.43 | 9.86 |
| YFP transc. trained axons rep 1 | 9.6 | 29,436,121 | 29,436,121 | 92.82 | 88.19 | 9.30 |
| YFP transc. trained axons rep 2 | 9.1 | 33,504,177 | 33,504,177 | 95.48 | 87.93 | 9.49 |
| YFP transc. trained cortex rep 1 | 9.4 | 33,113,755 | 33,113,755 | 95.15 | 87.57 | 9.53 |
| YFP transc. trained cortex rep 2 | 9.6 | 31,485,033 | 31,485,033 | 94.04 | 87.87 | 9.57 |

RIN: RNA Integrity Number; Q =-10 x log_10_(*p*) where *p*=probability of incorrect base call
